# Assessing the association between floods and dengue incidence in Vietnam: A time series study

**DOI:** 10.1097/EE9.0000000000000507

**Published:** 2026-07-21

**Authors:** Tu Nguyen Tien Tue, Xerxes Seposo, Hong Tam Nguyen, Hung Nguyen Viet, Nhung Hong Ha, Hai Tuan Nguyen, Nghia Ngu Duy, Lina Madaniyazi

**Affiliations:** a School of Tropical Medicine and Global Health, Nagasaki University, Nagasaki, Japan; b Ho Chi Minh City Center for Disease Control, Ho Chi Minh City, Vietnam; c School of Medicine, Hokkaido University, Hokkaido, Japan; d Ateneo Center for Research and Innovation, Ateneo School of Medicine and Public Health, Ateneo de Manila University, Philippines; e Ho Chi Minh City Infrastructure Management Center, Ho Chi Minh City, Vietnam; f National Institute of Hygiene and Epidemiology, Hanoi, Vietnam

## Abstract

**Background:**

Dengue incidence has increased significantly in recent decades, especially in areas undergoing rapid urbanization and frequent flooding. Floods can promote dengue transmission by creating favorable mosquito breeding sites, yet the timing and strength of this association remain unclear, particularly in Vietnam, where flood-prone areas overlap with a high dengue burden.

**Methods:**

This study assessed the relationship between flood exposure and dengue incidence across Vietnam and in Ho Chi Minh City (HCMC), a densely populated, flood-prone urban center. Time-series analyses were conducted using monthly data from 63 provinces (2011–2019) and weekly data from 24 districts in HCMC. Generalized additive mixed models with a negative binomial distribution were used, incorporating distributed lag linear functions to estimate cumulative and delayed effects. Analyses were stratified by season (rainy vs. dry).

**Results:**

At the national level, flooding was associated with a 17% higher dengue incidence (relative risk [RR] = 1.17; 95% confidence interval [CI] = 0.94, 1.47), with the strongest effect observed 2 months after flooding (RR = 1.11; 95% CI = 1.03, 1.20). Associations were more pronounced during the rainy season (RR = 1.65; 95% CI = 1.17, 2.32). In HCMC, floods were linked to a 10.4% increase in dengue cases (RR = 1.10; 95% CI = 1.03, 1.18), with effects peaking at week 9 (RR = 1.03; 95% CI = 1.02, 1.04). Seasonal analysis revealed short-term declines in risk during the rainy season and delayed increases during the dry season, peaking at week 9 (RR = 1.03; 95% CI = 1.01, 1.04).

**Conclusions:**

These findings highlight the delayed and seasonal nature of flood-related dengue risk and support the integration of flood metrics into early warning systems and climate-informed vector control strategies.

**What this study adds**

This is the first study to quantify the association between floods and dengue using nationwide data from Vietnam alongside high-resolution district-level analysis in Ho Chi Minh City. It reveals that flood exposure is followed by delayed increases in dengue incidence, peaking 2−3 months postflood. These findings highlight that dengue risk does not rise immediately after flooding but unfolds over time, underscoring the need for sustained public health preparedness. The study strengthens the case for integrating flood risk into dengue control and climate adaptation strategies in Southeast Asia and other flood-prone, dengue-endemic regions.

## Introduction

Dengue fever is one of the fastest-growing mosquito-borne diseases worldwide, transmitted primarily by *Aedes aegypti* and, to a lesser extent, *Aedes albopictus*. The World Health Organization (WHO) reported that the number of reported dengue cases increased from approximately 500,000 in 2000 to 5.2 million in 2019.^1^ Model-based estimates further suggest that dengue causes approximately 390 million infections annually, of which around 96 million are clinically apparent.^2^ Endemic to tropical and subtropical regions, dengue represents a major and growing public health challenge.^1,3^

Floods are among the most frequent and damaging natural disasters and may influence dengue transmission through multiple pathways. Floodwaters can create new mosquito breeding habitats, overwhelm drainage systems, and alter human-vector contact patterns. Conversely, heavy rainfall and flooding may initially reduce vector abundance through the flushing of mosquito larvae from breeding sites. As a result, the effects of flooding on dengue transmission are often complex and may occur with substantial delays following a flood event.^4^

Existing epidemiological studies have reported heterogeneous findings, with flood-dengue associations varying across geographic settings, climatic conditions, and study designs.^4^ For example, several studies have reported increased dengue incidence in the weeks following flood events,^5,6^ whereas others reported limited or inconsistent evidence of an association.^7,8^ This variability reflects the complex interplay of environmental conditions, mosquito ecology, human behavior, and broader social factors. As a result, the association between flooding and dengue remains incompletely understood, particularly in highly vulnerable settings.

Vietnam is one such setting. It is consistently ranked among the world’s most flood-exposed countries, with large coastal populations, densely populated deltas, and urban centers at risk of both riverine and coastal flooding.^9–11^ At the same time, dengue remains hyperendemic and represents a major public health challenge in Vietnam. Reported annual cases ranged from approximately 80,000–184,000 between 2015 and 2018, while the major outbreak in 2019 exceeded 320,000 cases and accounted for approximately 6.15% of global dengue cases.^12,13^ Dengue also imposes a substantial economic burden, estimated at approximately US$94.87 million annually (2016 prices).^14^ Despite the co-occurrence of recurrent flooding and substantial dengue burden, the epidemiological relationship between flooding and dengue in Vietnam remains poorly characterized.

This study aims to quantify the magnitude and timing of flood-associated dengue risk in Vietnam. We first conducted a nationwide analysis and subsequently focused on Ho Chi Minh City (HCMC), Vietnam’s largest city, with a population exceeding 9 million in 2019 (approximately 9.4% of the national population).^15^ As a densely populated, highly urbanized, and flood-prone setting that experiences recurrent dengue outbreaks, HCMC provides an important context in which to examine flood-dengue associations at a finer spatial and temporal scale. By characterizing the relationship between flooding and dengue incidence at both national and sub-national levels, this study addresses an important evidence gap regarding the role of flooding in dengue transmission in Vietnam.

## Methods

We collected data on dengue incidence, flood exposure, mean temperature, total precipitation, and population-socioeconomic factors across 63 provinces in Vietnam, as well as 24 districts in HCMC (Figure S1; https://links.lww.com/EE/A446). Time-series analyses were conducted to assess the association between flood exposure and dengue incidence at the provincial-month level nationwide and at the district-week level in HCMC. The study periods (2011–2019 for the national analysis and 2011–2017 for HCMC) were selected based on the availability of complete and validated datasets. The province-level analysis across Vietnam was restricted to the pre-COVID-19 period to avoid potential bias arising from pandemic-related disruptions to healthcare-seeking behavior, disease surveillance, population mobility, and vector control activities.

### Dengue data collection and definition

Monthly dengue case data for all 63 provinces in Vietnam from 2011 to 2019 were obtained from the Southeast Asia Research on Climate Change and Dengue platform (https://sites.google.com/view/searcd/home?authuser=0), a regional initiative that harmonizes dengue surveillance data. For Vietnam, these data were provided by the National Institute of Hygiene and Epidemiology, the country’s leading public health research institute. For the district-level analysis across HCMC, weekly dengue surveillance data for each of the city’s 24 districts from 2011 to 2017 were obtained from the Ho Chi Minh City Center for Disease Control.

For both national and HCMC datasets, dengue cases were defined according to national surveillance standards. Reported cases represent patients who met the case definition for dengue and were recorded by date of symptom onset, while reported deaths represent those confirmed to have died from dengue, recorded by date of death. In this study, fatal cases were not counted separately; all analyses were based on the total number of reported dengue cases. Therefore, “dengue cases” refers to all unique individuals recorded in the national surveillance system, irrespective of clinical outcome, including both recoveries and deaths. Further details on the organizational structure of surveillance and case definitions are provided in the Supplementary Material; https://links.lww.com/EE/A446.

### Flood data collection and definition

For the province-level analysis, flood data from 2010 onward were obtained from the National Steering Committee for Natural Disaster Prevention and Control (NSCNDPC; hereafter NSC). The Standing Office of the NSC issues daily reports on flood situations, including river water levels at key stations, comparisons with alarm thresholds, flood progression, and documented impacts on people, housing, infrastructure, crops, and transportation. These reports are derived from summary reports submitted by individual provinces and are publicly available.^16^

For the district-level analysis across HCMC, flood data were obtained from the HCMC Infrastructure Management Center through a formal application and approval process. HCMC Infrastructure Management Center maintains detailed records of localized urban flooding caused by heavy rainfall and tidal inundation, collected through a combination of water-level sensors, CCTV surveillance, flood mapping systems, and field observations. Notably, these records are not routinely integrated into the NSC database and thus provide unique information for HCMC at the district level.

For this study, flood exposure was defined as a binary exposure variable. For the province-level analysis, a flood was recorded if at least one flood event occurred in a province during a given month, based on NSC reports. Terms such as “flood,” “inundation,” “submergence,” “flash flood,” “river/riverine flood,” and “stream flood” were used to identify events. Floods caused by seawater intrusion or sea-level rise were excluded, as saline conditions are unsuitable for the life cycle of *Aedes* mosquitoes.^17^ For the district-level analysis across HCMC, a flood was recorded if at least one event occurred in a district during a given week.

### Weather, population, socioeconomic, and land use data

Weather information was included to account for potential confounding, as dengue transmission is strongly influenced by temperature and precipitation.^5,8,18,19^ Mean temperature (°C) and total precipitation (mm) were obtained from the ERA5-Land dataset of the European Centre for Medium-Range Weather Forecasts Climate Data Store, which provides hourly climate reanalysis data at a spatial resolution of 0.1° × 0.1° (approximately 9 km).^20^

Weather variables were extracted using an area-weighted approach and aggregated into monthly averages and totals for each province from 2011 to 2019, and into weekly values for each district in HCMC from 2011 to 2017. Although temporal aggregation may smooth short-term meteorological fluctuations and extreme events, it ensured consistency with the temporal resolution of the epidemiological data. Details of the extraction and aggregation procedures are provided in the Supplementary Material; https://links.lww.com/EE/A446.

To assess consistency with an alternative gridded climate product, we compared ERA5-Land temperature and precipitation estimates with the WorldClim 2.1 (CRU-TS 4.09) dataset across Vietnam’s 63 provinces over the study period.

Population, socioeconomic, and land use data were obtained from the Statistical Yearbooks published by the General Statistics Office of Vietnam at the national level and by the Ho Chi Minh City Statistics Office (HSO) at the city level.^15,21^ These annual data include population size and density, poverty rate, and average income per capita, as well as land use classifications (agricultural production land, forestry land, special-use land, and residential land). These indicators were selected based on their documented relevance to flood risk and dengue transmission.^22–32^ Although they may act as potential confounders, they were not included in the primary model to preserve parsimony and avoid adjustment, given their relatively slow temporal variation and correlation with spatial random effects. Instead, they were incorporated as adjustment covariates in sensitivity analyses to assess the robustness of the estimated flood-dengue association. Additional details are provided in the Supplementary Material; https://links.lww.com/EE/A446.

### Statistical analysis

Before analysis, the dataset was screened for duplicate records, missing values, and potential outliers. No duplicates or missing values were identified, and all apparent outliers were verified against the original sources and retained. Consequently, the final dataset was complete for the study period.

We used generalized additive mixed models (GAMMs) with a negative binomial distribution to estimate the association between flood presence and dengue incidence by accounting for overdispersion in count data, spatial heterogeneity, and long-term temporal trends.

Smooth spline functions of time were used to control for long-term trends and seasonality (3 degrees of freedom (*df*) per year for the province-level analysis across Vietnam and 5 *df* per year for the district-level analysis across HCMC). Mean temperature and total precipitation were included as covariates using natural cubic splines with 3 *df*. For the province-level analysis across Vietnam, temperature and precipitation were averaged over the preceding 3 months; for the district-level analysis across HCMC, they were averaged over the preceding 12 weeks.

Flood exposure was modeled using a cross-basis function to capture both immediate and delayed effects. The exposure–response function was specified as linear, while the lag–response relationship was modeled using natural cubic splines with 3 *df*. The lag structure was set to 0–4 months for the province-level analysis across Vietnam and 0–16 weeks for the district-level analysis across HCMC. These lag windows were selected a priori based on the biologically plausible delay between flooding and reported dengue cases, including the time required for postflood mosquito breeding habitat formation, mosquito development, viral incubation within the vector, human infection, symptom onset, and case notification. This timeframe is also consistent with previous flood-dengue studies,^5,6^ which evaluated delayed effects within approximately 1–4 months following flood events. The 0–16-week lag used in the HCMC analysis corresponds to the 0–4-month window used in the national analysis while accounting for the finer weekly temporal resolution of the HCMC dataset.

Models included an offset term for population size to account for variation in the population at risk. Random intercepts at the province (national analysis) or district (HCMC analysis) level were included to account for unmeasured spatial heterogeneity. Stratified analyses were conducted for the dry (November–April next year) and rainy (May–October) seasons. Seasonal classifications followed the National Technical Regulation on Natural Condition Data for Construction.^33^

### Sensitivity analysis

Several sensitivity analyses were conducted. First, additional population, socioeconomic, and land-use variables were incorporated using a forward stepwise, cumulative approach, whereby each successive model retained all covariates from the preceding model and added one additional variable. Second, we repeated the main analyses using alternative lag windows for flood exposure and varying *df* for the natural cubic splines used to model long-term trends and seasonality. Third, for the province-level analysis across Vietnam, we repeated the analysis using population-weighted temperature and precipitation estimates in place of the primary area-weighted meteorological variables to evaluate the robustness of the findings to alternative representations of meteorological exposure.

The full model specification and sensitivity analyses are described in the Supplementary Material; https://links.lww.com/EE/A446. All data processing and analyses were performed using R (version 4.3.2) and R Studio (version 2025.05.1 + 513).

## Results

Between 2011 and 2019, a total of 1,129,083 dengue cases and 1,146 flood months were reported across 63 provinces in Vietnam (Table S1; https://links.lww.com/EE/A446). All provinces experienced at least one flood during the study period, with an average of 53 provinces affected annually. In HCMC, 121,165 dengue cases and 1,987 flood weeks were recorded across 24 districts during 2011–2017 (Table S2; https://links.lww.com/EE/A446), with flooding reported in 22 of the 24 districts. Dengue incidence showed a clear seasonal pattern, with higher case counts from July to November and peak outbreaks consistently occurring between August and October (Figures S2 and S3; https://links.lww.com/EE/A446). Flood exposure also exhibited seasonality, occurring most frequently in September and October (Figures S2 and S3 https://links.lww.com/EE/A446).

Summary statistics for temperature, precipitation, and other covariates are provided in the Supplementary Material (Tables S1–S2 and Figures S4–S7; https://links.lww.com/EE/A446). A supplementary comparison between ERA5-Land and WorldClim 2.1 showed high agreement for temperature and moderate agreement for precipitation across Vietnam (Table S3; https://links.lww.com/EE/A446).

**Figure 1.**
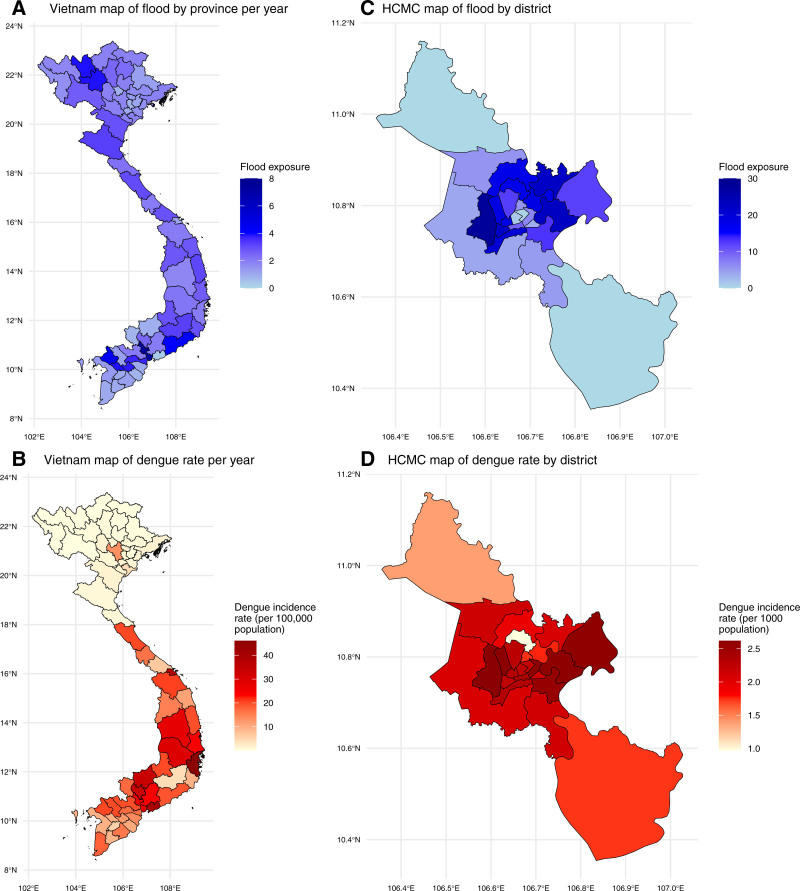
Annual exposure to flood and annual incidence rate of dengue by province in Vietnam and Ho Chi Minh City (HCMC). Flood exposure was defined as a binary exposure variable. At the national level, a flood was recorded if at least one flood event occurred in a province during a given month. At the HCMC level, a flood was recorded if at least one event occurred in a district during a given week.

Flood exposure was most frequent in northern and central provinces, while dengue incidence was highest in the south, with Hanoi an exception among northern regions (Figure 1). HCMC recorded both the highest flood intensity and the largest dengue burden. Within HCMC, flood events were concentrated in central districts, which also exhibited higher dengue incidence (Figure 1).

### Province-level analysis across Vietnam

**Figure 2.**
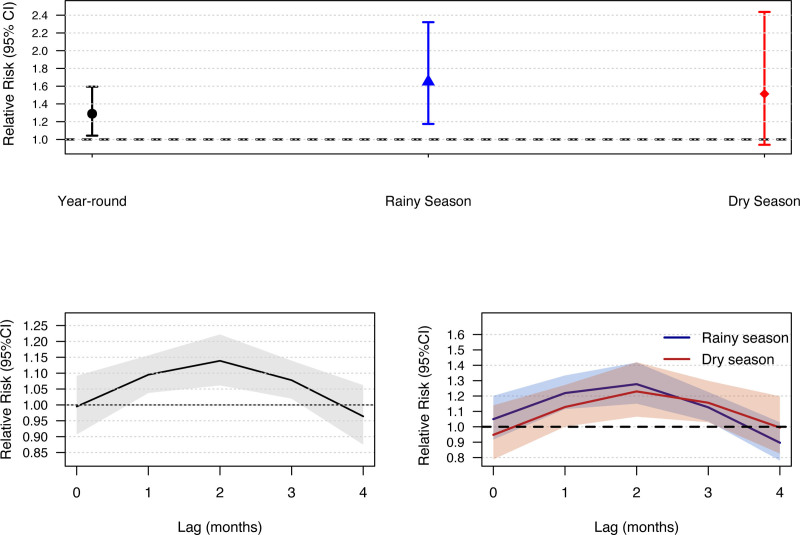
Association between flood exposure and dengue incidence in Vietnam, analyzed year-round (black), during the rainy season (blue), and the dry season (red). The top panel presents the cumulative exposure–response association, while the bottom two panels illustrate the lag–response relationships over time. RR (95% CI): relative risk with corresponding 95% confidence interval.

Floods were associated with a 17% higher dengue incidence nationally (relative risk [RR] = 1.17; 95% confidence interval [CI] = 0.94, 1.47). The lag-response relationship showed no evidence of an immediate effect in the same month (lag 0: RR = 0.96; 95% CI = 0.87, 1.06). Dengue risk increased 1 month after flood exposure (lag 1: RR = 1.06; 95% CI = 1.00, 1.13), peaked at 2 months postexposure (lag 2: RR = 1.11; 95% CI = 1.03, 1.20), and declined at 3 months (lag 3: RR = 1.07, 95% CI = 1.01, 1.13) before hovering to the null by the fourth month (lag 4: RR = 0.97, 95% CI = 0.88, 1.07) (Figure 2).

During the rainy season, there were 922 instances of flood exposure, compared with 305 in the dry season. Seasonal analyses indicated stronger associations in the rainy season. The RR was 1.65 (95% CI = 1.17, 2.32) compared with 1.51 (95% CI = 0.94, 2.44) in the dry season. In rainy months, risk increased from 1 month after floods, peaking at 2 months (RR = 1.28, 95% CI = 1.15, 1.42), and persisting into the third month. In dry months, a weaker delayed effect was observed, also peaking 2 months after floods (RR = 1.23; 95% CI = 1.06, 1.43).

### District-level analysis across HCMC

**Figure 3.**
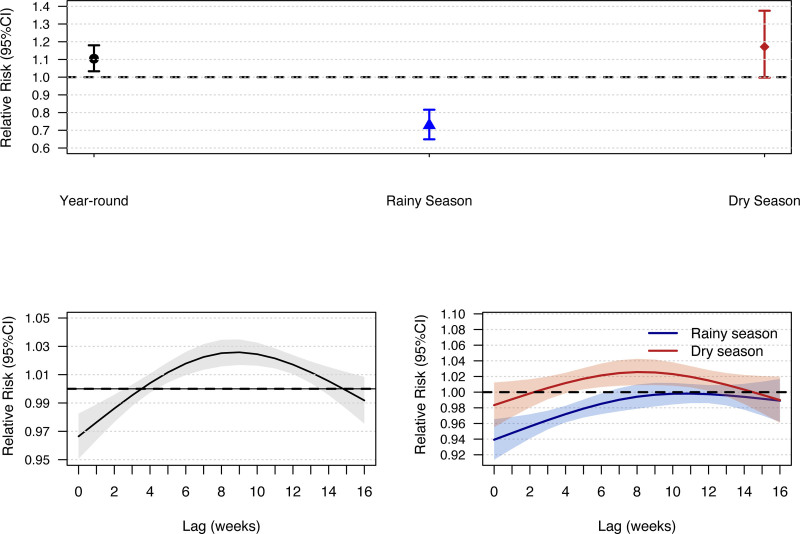
Association between flood exposure and dengue incidence in Ho Chi Minh City, analyzed year-round (black), during the rainy season (blue), and the dry season (red). The top panel presents the cumulative exposure–response association, while the bottom two panels illustrate the lag–response relationships over time. RR (95% CI): relative risk with corresponding 95% confidence interval.

In HCMC, the results showed a 10.4% higher dengue incidence across 16 weeks (RR = 1.10; 95% CI = 1.03, 1.18). Lag-response associations indicated a short-term reduction during and immediately after floods (lags 0–2 weeks, all RRs <1). From week 5 to week 13, incidence increased steadily, peaking at week 9 (RR = 1.03, 95% CI = 1.02, 1.04), before hovering back to the null (Figure 3).

Seasonal analyses showed contrasting patterns. During the rainy season, flooding was associated with consistently lower dengue incidence, with RRs remaining below 1 up to lag 6 weeks, before returning to null values. In contrast, during the dry season, flooding was associated with an increased risk, with an RR of 1.17 (95% CI = 1.00–1.38). Lag-specific effects showed no notable change in the first 4 weeks postflooding, followed by an increased incidence between weeks 5 and 12, reaching a peak at week 9 (RR = 1.03, 95% CI = 1.01, 1.04).

Our estimates from both the national and HCMC analyses remained robust in sensitivity analyses (Tables S4–S8 https://links.lww.com/EE/A446).

## Discussion

This study provides national and city-level evidence on the association between flooding and dengue incidence in Vietnam. Using time-series analyses, we observed delayed increases in dengue incidence following flood exposure at both national and HCMC levels. In the province-level analysis across Vietnam, the strongest association was observed 2 months after flooding, with elevated risks persisting through the third month. In the district-level analysis across HCMC, dengue incidence was approximately 10% higher following flood exposure over the subsequent 16 weeks, with incidence declining during the first 2 weeks before rising from week 3 to week 13 and peaking at week 9 (approximately 2 months after flooding).

Several studies have examined the relationship between flooding and dengue.^4–8,34^ Reported associations have varied across settings, ranging from increased dengue incidence following floods to weak, inconsistent, or context-dependent associations. However, direct comparisons are challenging because “flood exposure” has been defined inconsistently across studies. For example, Carvajal et al^5^ defined flood conditions as instances where major roads became impassable due to inundation and incorporated this indicator into dengue forecasting models in Manila, Philippines.^5^ Lowe et al^34^ characterized flood-prone conditions using a combination of long-term precipitation and short-term wet conditions, whereas Liu et al^7^ examined annual measures of flood duration and frequency. These differences in exposure definition likely capture distinct dimensions of flooding, including physical inundation, hydrometeorological conditions conducive to flooding, and broader population-level flood burden. As a result, estimated associations may not be directly comparable across studies. In the present study, flood exposure was defined using officially reported flood events from national disaster management records, providing a consistent and operationally relevant measure that could be applied across all provinces and years included in the analysis. Taken together, these findings suggest that observed flood–dengue associations may vary not only across geographic settings but also according to how flood exposure is defined. The existing evidence indicates that flooding can influence dengue transmission, although the magnitude, timing, and direction of this relationship appear to depend on local environmental and epidemiological conditions.

Our findings suggest that the impact of flooding on dengue is delayed rather than immediate. In the province-level analysis across Vietnam, the strongest association was observed approximately 2 months after flooding, while in the district-level analysis across HCMC, the peak effect occurred at week 9. These findings are broadly consistent with previous studies. Lowe et al^34^ reported that dengue outbreaks were most likely to occur approximately 1 month after excessive rainfall, with the highest RR observed at lags of 1–2 months. Similarly, Carvajal et al^5^ identified flood exposure at a 6-week lag as an important predictor of dengue incidence, while a recent machine-learning study^35^ reported the strongest predictive effects of flood-related indicators at lags of approximately 5–7 weeks. Collectively, these findings suggest that flood-related increases in dengue risk typically emerge several weeks after flooding events.

The absence of an immediate increase in dengue risk may reflect several mechanisms, including a hypothesized flushing effect, whereby heavy rainfall and floodwaters temporarily wash away mosquito larvae and pupae, disrupting breeding habitats.^4,8,36^ However, this interpretation should be considered alongside alternative explanations. For example, flooding may temporarily disrupt healthcare access and disease surveillance activities, leading to delays in case detection and reporting. In addition, intensified postflood vector control measures could temporarily suppress mosquito populations and reduce transmission. As floodwaters recede and stagnate, however, they create favorable conditions for vector reproduction. Combined with the mosquito life cycle (7–10 days), the extrinsic incubation period (mean 6.5–15 days depending on temperature), and the intrinsic incubation period (mean 5.9 days),^37,38^ and the additional time from infection to diagnosis, this provides a biologically plausible explanation for the observed delayed increase in dengue incidence.

Seasonal analyses showed increased dengue risk following floods during both the rainy and dry seasons in the province-level analysis across Vietnam. In contrast, the district-level analysis in HCMC suggested a reduction in dengue risk following flood exposure during the rainy season. One possible explanation for this discrepancy is that the impact of flooding may depend on the surrounding environmental and urban context. At the national level, rainy-season conditions, including high humidity, warm temperatures, and abundant water availability, are generally favorable for mosquito survival and reproduction, potentially amplifying the effects of flooding on dengue transmission. In highly urbanized settings such as HCMC, however, floodwaters may drain more rapidly and be accompanied by cleanup activities or vector control interventions. In addition, intense rainfall and flooding may reduce mosquito abundance through a flushing–drying mechanism, whereby larvae are washed from breeding sites and subsequent drying limits the formation of suitable aquatic habitats. Supporting this hypothesis, studies conducted in urban Singapore found that monsoon-driven flushing and drying events substantially reduced Aedes aegypti breeding and were associated with a lower risk of dengue outbreaks for up to 6 weeks after the events occurred.^8,36^ Postflood cleanup activities and vector control measures may further contribute to temporary reductions in transmission. By contrast, the positive associations observed during the dry season may reflect the persistence of stagnant water in urban environments following flood events, creating breeding habitats that support subsequent mosquito proliferation and dengue transmission. Nevertheless, further entomological studies are needed to evaluate these mechanisms directly.

A notable strength of this study is the use of multi-year, multi-province data to quantify the association between flood events and dengue incidence in Vietnam, a country frequently affected by both flooding and dengue, yet with limited research examining how these phenomena may interact. In addition, the analysis also incorporated weekly data at the district level in HCMC, allowing for a more detailed assessment of spatial and temporal variations in flood-related dengue risk within a more localized administrative setting. The findings of this study can contribute valuable evidence, offering insights that are especially relevant for public health preparedness in flood-prone, dengue-endemic regions. The identified lag periods may provide a useful window for targeted public health interventions, including intensified vector control, community risk communication, and enhanced dengue surveillance following flood events.

Several limitations must be acknowledged. First, the ecological nature of this study limits the capacity to reflect individual-level risk patterns within affected populations. Since the analysis relies on aggregated data across provinces and years, it cannot account for individual exposures or behavioral factors that may influence dengue risk. Consequently, causal inference is restricted, and findings should be interpreted with caution when informing targeted interventions. Second, the flood data sources differed between the national and city-level analyses. National-level flood data are derived from summary reports submitted by individual provinces to the National Steering Committee. These reports vary in format, completeness, and level of detail, often lacking standardized event-level documentation. In contrast, flood records in HCMC are systematically maintained and event-specific, enabling relatively more detailed and consistent exposure assignment. As a result, exposure measurement error may differ across study settings, and direct comparisons of effect estimates between the national and HCMC analyses should be interpreted cautiously. In addition, flood exposure was defined as the occurrence of at least one reported flood event within a province-month (or district-week in HCMC), representing the most complete and consistent metric available across the study period. However, this binary definition does not capture potentially important dimensions of flooding, including event frequency, duration, depth, severity, or spatial extent. Consequently, some exposure variability may not have been captured, limiting the ability to assess dose–response relationships and potentially affecting the precision and magnitude of the estimated associations. Although more detailed flood metrics could potentially be derived from satellite-based remote sensing and hydrological monitoring systems, these approaches remain subject to uncertainties in flood detection, classification, and validation. Moreover, the resulting metrics may not be directly comparable with the official historical flood records used in this study. We therefore relied on nationally available flood reports, which provided a consistent and reproducible source of exposure information across provinces and throughout the study period. Nevertheless, we considered this approach preferable to incorporating incomplete or inconsistently reported severity measures that could introduce additional measurement error and reduce comparability across provinces and years. Lastly, several unmeasured confounding factors could not be explicitly accounted for in our models. Variations in population mobility, local vector control intensity, and the quality of urban infrastructure, such as drainage efficiency and housing density, are likely to influence local transmission dynamics. The direction of bias introduced by these factors is difficult to predict, as they may be associated with both flood exposure and dengue risk in complex and potentially opposing ways.

Future research should prioritize the development of standardized flood monitoring systems and more refined exposure metrics incorporating flood duration, depth, and spatial extent, where data quality allows. Methodological advances integrating hydrological, entomological, and epidemiological data may further improve understanding of the timing, magnitude, and mechanisms of flood-related dengue risk and help identify opportunities for more targeted public health interventions.

## Conclusion

This study provides nationwide and city-level evidence on the association between flood and dengue in Vietnam. In the province-level analysis across Vietnam, flood exposure was associated with increased dengue incidence 2–3 months after flooding, with the strongest association observed at two months (RR = 1.11, 95% CI = 1.03–1.20). In the district-level analysis across HCMC, flood exposure was associated with a 10% increase in dengue incidence over the subsequent 16 weeks (RR = 1.10, 95% CI = 1.03–1.18), with the peak effect occurring at week 9.

A key finding was that flood-related dengue risk does not occur immediately but emerges after a delay, peaking weeks to months after flooding. These findings suggest that public health preparedness and dengue surveillance may benefit from extending beyond the immediate aftermath of flood events. However, the results should be interpreted in light of several limitations, including the ecological study design and the use of simplified flood exposure measures that did not capture flood severity, duration, or spatial extent. Future studies integrating standardized flood monitoring systems with high-resolution hydrological, entomological, and epidemiological data may help clarify the mechanisms underlying flood–dengue relationships and improve risk assessment in flood-prone settings.

## Conflict of interest statement

The authors declare that they have no conflicts of interest with regard to the content of this report.

## Supplementary Material


