# Supplementary material for: Assessing the association between floods and dengue incidence in Vietnam: A time series study
Source: Environ Epidemiol. 2026 Jul 21;10(4):e507. doi: 10.1097/EE9.0000000000000507 (PMC13391126; doi:10.1097/EE9.0000000000000507)
Supplement: Supplementary file 1 [file ee9-10-e507-s001.pdf]

Supplementary Material

Assessing the association between floods and dengue incidence in Vietnam: A time series study

Nguyen Tien Tue Tu[1,2], Xerxes Seposo[1,3,4], Nguyen Hong Tam[2], Nguyen Viet Hung[5], Ha Hong Nhung[6], Nguyen Hai Tuan[6], Ngu Duy Nghia[6], Lina Madaniyazi[1*]

1. School of Tropical Medicine and Global Health, Nagasaki University, Nagasaki
2. School of Medicine, Hokkaido University, Nagasaki
3. Ateneo Center for Research and Innovation, Ateneo School of Medicine and Public Health, Ateneo de Manila University, Philippines
4. Ho Chi Minh City Center for Disease Control
5. Ho Chi Minh City Infrastructure Management Center
6. National Institute of Hygiene and Epidemiology, Hanoi, Vietnam

*** Corresponding author**

Lina Madaniyazi, lina.madaniyazi@nagasaki-u.ac.jp

School of Tropical Medicine and Global Health, Nagasaki University,

Nagasaki 852-8523, Japan

1. Organizational structure of surveillance and case definitions of dengue in Vietnam

For dengue data in Vietnam, members from the National Institute of Hygiene and Epidemiology (NIHE), which is the leading public health research institute in the country, have been providing data for the platform. Established in 1945, NIHE operates under the Ministry of Health and carries various responsibilities, including conducting epidemiological studies, collaborating with international organizations to address global health challenges, and enhancing public health outcomes in Vietnam and the surrounding region. The data for this study were obtained through an application and approval process from SEARCD collaborators.

Dengue cases and deaths in each province were reported to the Center for Disease Control of the given province by hospitals, clinics, commune health stations, district health centers, and other health facilities within the province, in accordance with the guidelines outlined in Circular No. 48/2010/TT-BYT issued by the Ministry of Health for reporting infectious diseases (Figure below).[1] Therefore, in HCMC, the weekly dengue surveillance data for this study were obtained from the Ho Chi Minh City Center for Disease Control (HCDC) through a formal application and approval process. In the HCDC weekly reports, indicators were recorded by calendar week of each year, spanning from 00:00 on Monday to 24:00 on Sunday of the reporting week. The indicators include: (1) ***Number of cases***: The number of patients who meet the case definition for surveillance, recorded based on the date of symptom onset during the reporting period. (2) ***Number of deaths due to the disease:*** The number of deaths confirmed to be caused by the disease, recorded based on the date of death during the reporting period. (3) ***Cumulative cases and cumulative deaths***: The total number of cases and the total number of deaths accumulated over a specified surveillance period.

In this study, numbers of cases and deaths were aggregated and referred to as dengue cases.

Figure. Organizational structure for case reporting[1]

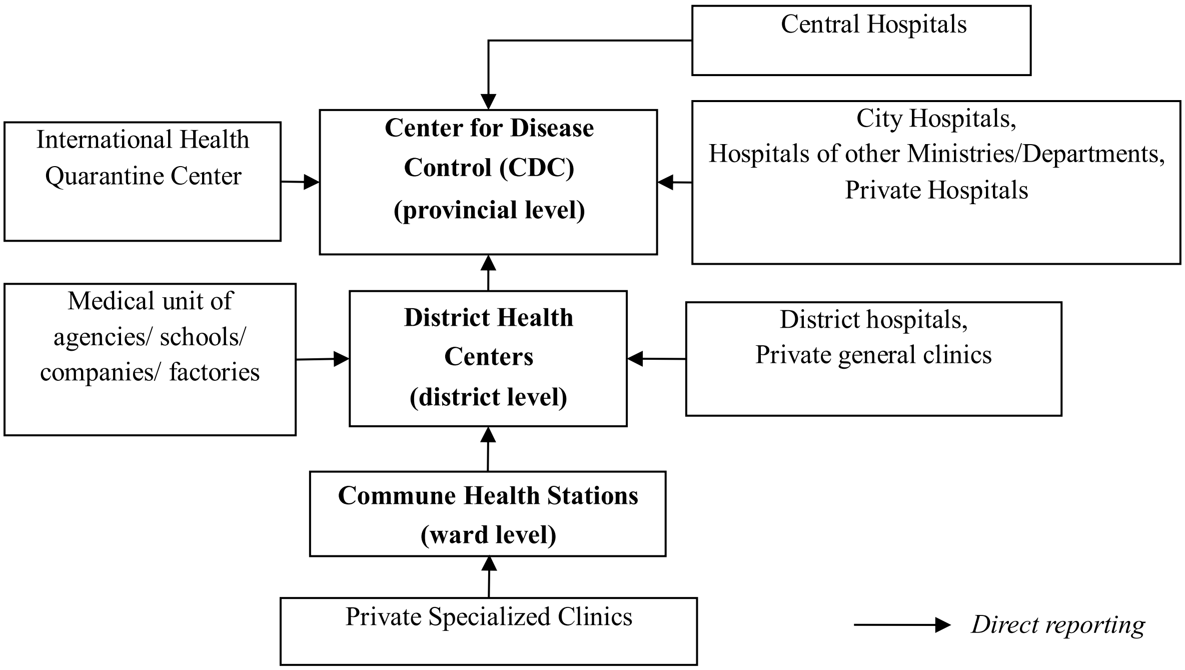

Definition of dengue cases

In Vietnam, dengue patient surveillance is guided by Decision No.1499/QD-BYT, issued by the Ministry of Health on May 17, 2011, which outlines protocols for surveilling and preventing dengue.[2] Although this Decision was replaced by Decision No.3711/QD-BYT issued on September 19, 2014, the case definition for surveillance in the guideline remained the same as follows:

- Suspected case (Clinical case): A person living in or coming from an area with dengue outbreaks or endemic areas within the past 14 days, having a sudden high fever lasting continuously for 2–7 days, along with at least two of the following symptoms:

- Bleeding manifestations, which can vary in severity, such as a positive tourniquet test, petechiae/ecchymosis under the skin, bleeding gums, or nosebleeds.
- Headache, loss of appetite, nausea, or vomiting.
- Flushed skin or rash.
- Muscle pain, joint pain, or pain behind the eyes.
- Restlessness or lethargy.
- Pain or tenderness in the liver area.

- Confirmed case: A case confirmed by laboratory diagnosis using techniques such as Mac-ELISA, PCR, NS1 antigen test, or virus.[3]

This definition in these guidelines was simplified and intended solely for surveillance purposes. A more detailed description and classification, such as dengue fever, dengue fever with warning signs, and severe dengue fever, are outlined in the "Dengue diagnostic and treatment guidelines" issued under Decision No.458/QD-BYT, dated February 16, 2011, by the Minister of Health.[4]

Periodic reports include details about dengue cases in the community and those who are examined and treated at public and private medical facilities. The reports specify the number of suspected cases and confirmed deaths in each administrative area based on the case definitions.

4. Statistical analysis

A generalized additive mixed model with a negative binomial distribution was used to evaluate the association between flood presence and dengue incidence. The model can be expressed as:

$$\log(Y_{it}) = a + ns(Time_t,\ df_t) + ns(Temp_{it}, df = 3) + ns(Precip_{it}, df = 3) \\ + cb(Flood_{it}, lag = LagRange, df = 3) + offset(\log(Pop_i)) + u_i$$

where:

- Y_{it} represents the number of dengue cases in administrative unit i at time t, where i refer to province and district in the national and HCMC analysis, respectively.
- a is the intercept term.
- $ns(Time_t, df_t)$ is a natural cubic spline function of continuous time, from 1 to 108 for the national level (first to last month of the study period), and from 1 to 365 for the HCMC level (first to last week of the study period), adjusting for long-term trends and seasonality.
    - For the national-level analysis, 3 degrees of freedom (df) per year was used.

    - For the HCMC-level analysis, 5 df per year was used.
- $ns(Temp_{it}, df = 3)$ is a natural cubic spline function adjusting for the average temperature over preceding periods, with df = 3, following the previous studies.[19,20]
    - For the national analysis, temperature was averaged over the previous 3 months.
    - For the HCMC analysis, temperature was averaged over the previous 12 weeks.
- $ns(Precip_{it}, df = 3)$ is a natural cubic spline function adjusting for the total precipitation over preceding periods, with df = 3, following the previous studies.[19,20]
    - For the national analysis, total precipitation was averaged over the previous 3 months.
    - For the HCMC analysis, total precipitation was averaged over the previous 12 weeks.
- $cb(Flood_{it}, lag = LagRange, df = 3)$ denotes the cross-basis function to model both the cumulative and lagged effects of flood presence.
    - The exposure-response relationship was specified as linear.
    - The lag-response relationship was captured by a natural cubic spline with 3 df.
    - The lag structure differed between analyses: 0–4 months for the national level and 0–16 weeks for the HCMC level.
- $offset(\log(Pop_i))$ was included as an offset term to account for varying population sizes across administrative units.
- u_i represents random effects at the administrative unit level (province for national-level, district for HCMC-level), controlling for unmeasured spatial heterogeneity.

The relative risk (RR) and its 95% confidence interval (CI) were used to estimate the effect of flood presence on dengue incidence. Model specifications, including the choice of functions, time of lag, and df, were informed by previous studies.[19,20]

Additionally, stratified analyses were conducted for dry and rainy seasons. In these models, the $ns(Time_t, df_t)$ was replaced by separate terms for *Year* and either *Month* of the year (for the national level) or *Week* of the year (for the HCMC level), respectively. *Month* or *Week* was treated as a categorical variable to account for recurring monthly or weekly seasonal effects, while *Year* was included as a linear term to capture long-term trends.

5. Figures

Figure S1. Location of HCMC in Vietnam[23]

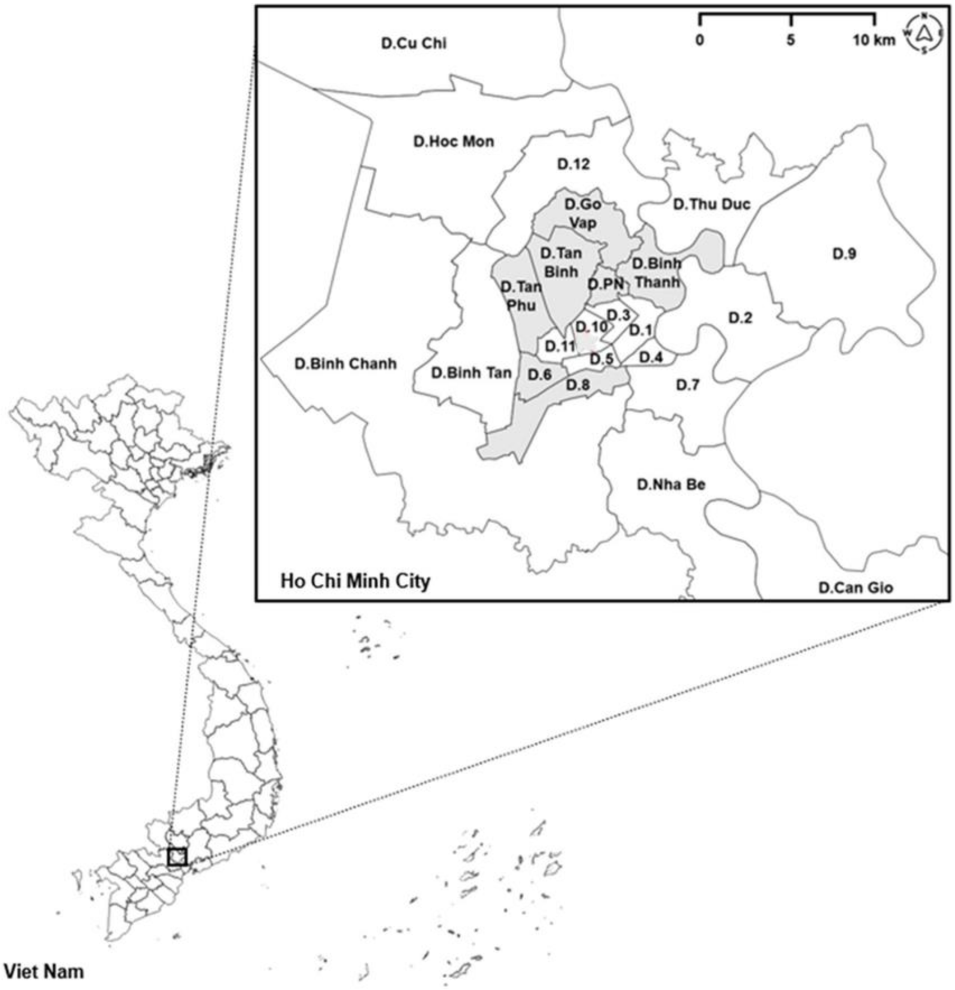

Figure S2. Monthly time series plot of flood exposure and dengue cases at the national level

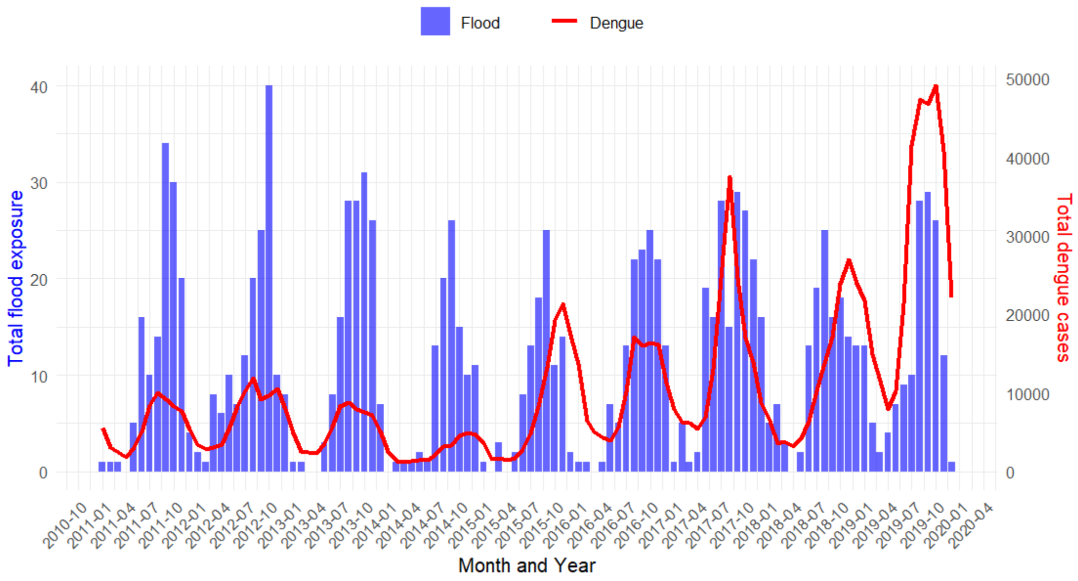

Figure S3. Weekly time series plot of flood exposure and dengue cases in HCMC

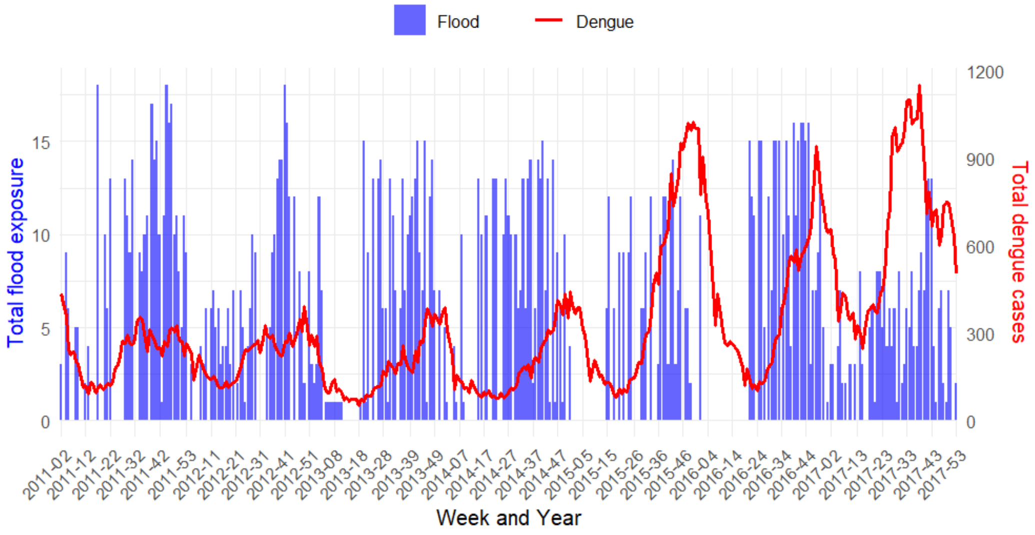

Figure S4. Time series plots of data on population, socioeconomic, and land use characteristics across 63 provinces in Vietnam

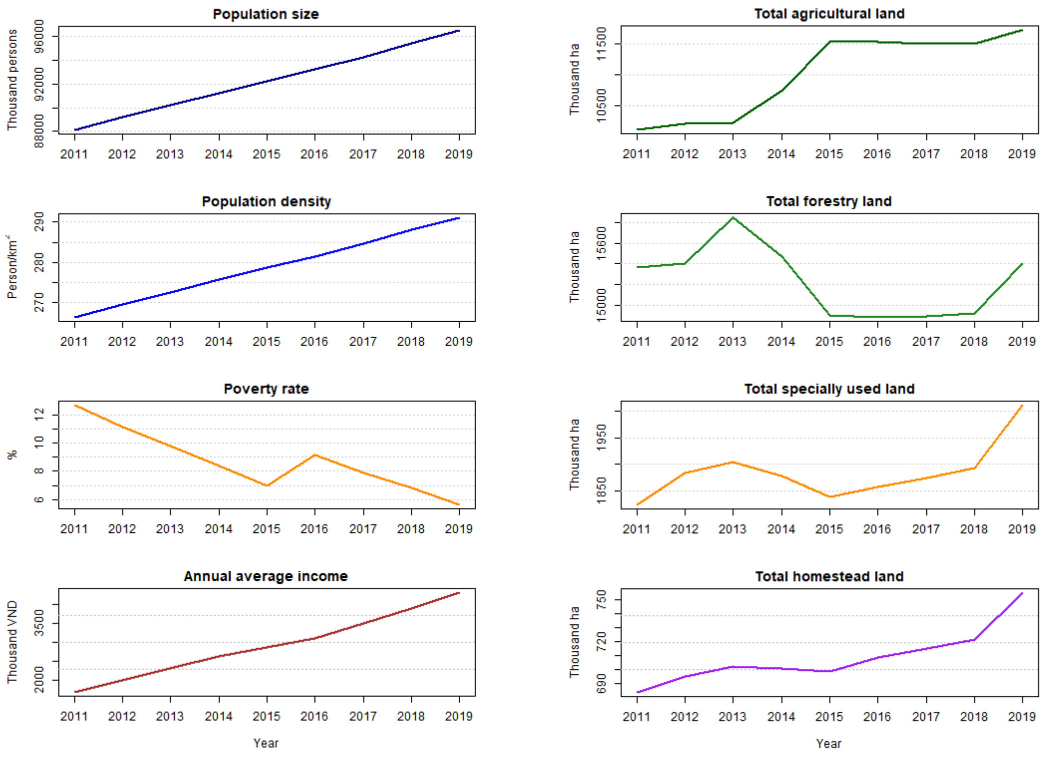

Figure S5. Distribution of population across provinces in Vietnam and districts in HCMC

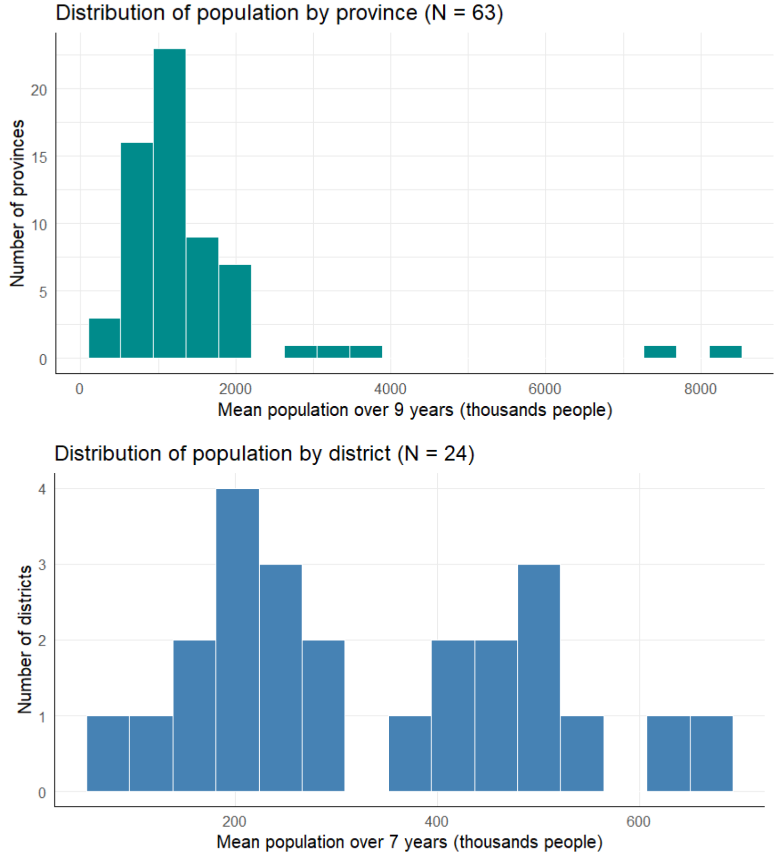

Figure S6. Distribution of land use types across provinces in Vietnam

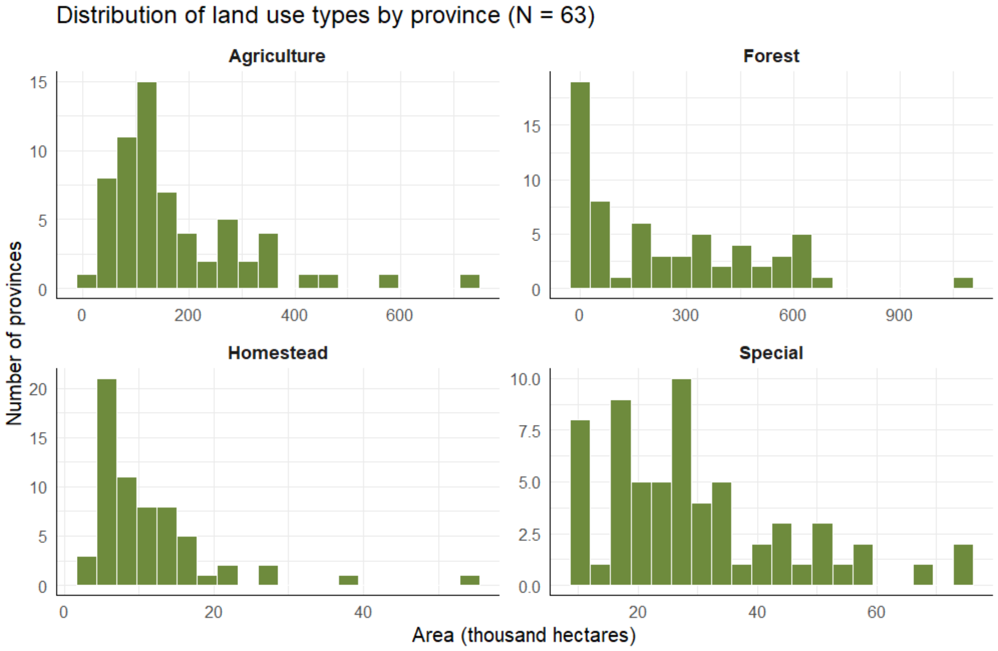

Figure S7. Time series plots of data on population and socioeconomic characteristics in HCMC

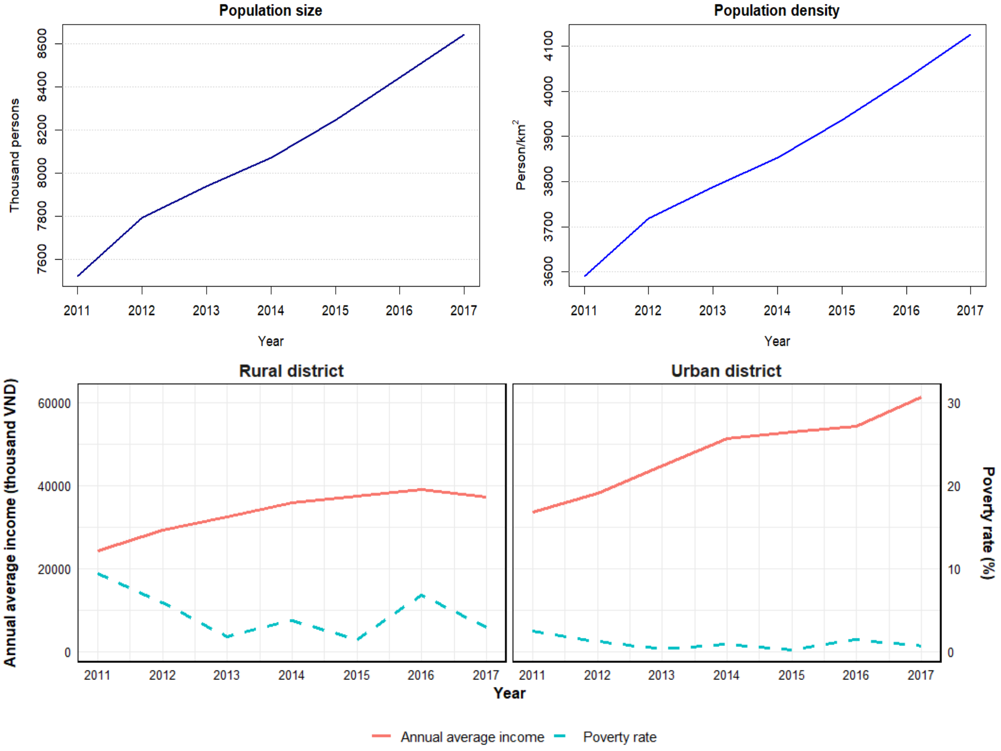

Note: Socioeconomic data for Ho Chi Minh City is not available at the individual district level. Instead, the districts are categorized as either urban or rural, and socioeconomic information is provided by the government for these two broad categories.

6. Tables

Table S1. Summary of monthly time series data on flood exposures, dengue cases, monthly mean temperature, and monthly total precipitation over 9 years for each province

Province	Flood exposure				Dengue count				Temperature (℃)		Precipitation (mm)	
	Total	Mean	Min	Max	Total	Mean	Min	Max	Mean	SD	Median	IQR
An Giang	39	0.36	0	1	36078	334.06	43	1256	27.13	1.08	75.53	86.90
Ba Ria - Vung Tau	3	0.03	0	1	40146	371.72	26	3895	26.58	0.93	40.61	55.84
Bac Giang	15	0.14	0	1	1469	13.60	0	401	22.65	4.82	51.46	83.69
Bac Kan	19	0.18	0	1	102	0.94	0	22	20.51	4.78	68.08	97.42
Bac Lieu	9	0.08	0	1	7235	66.99	3	266	26.88	0.96	65.96	65.01
Bac Ninh	12	0.11	0	1	2185	20.23	0	495	23.65	4.81	11.37	16.14
Ben Tre	8	0.07	0	1	15921	147.42	22	593	26.81	0.93	49.14	59.97
Binh Dinh	24	0.22	0	1	26249	243.05	4	1527	24.87	2.58	92.04	86.69
Binh Duong	21	0.19	0	1	65444	605.96	47	2663	27.06	1.13	56.41	78.55
Binh Phuoc	7	0.06	0	1	28876	267.37	10	1788	26.25	1.22	137.61	241.77
Binh Thuan	35	0.32	0	1	20899	193.51	11	1225	25.46	0.94	152.44	163.42
Ca Mau	9	0.08	0	1	19470	180.28	10	1468	26.94	0.94	136.94	120.59
Can Tho	34	0.31	0	1	10636	98.48	20	565	26.85	1.05	33.78	34.98
Cao Bang	14	0.13	0	1	57	0.53	0	12	19.67	4.94	86.07	103.83
Da Nang	10	0.09	0	1	37126	343.76	0	2835	24.54	2.87	23.65	18.12
Dak Lak	19	0.18	0	1	49080	454.44	4	6129	24.05	1.66	214.03	228.90
Dak Nong	24	0.22	0	1	10844	100.41	0	1336	23.24	1.31	125.57	169.15
Dien Bien	12	0.11	0	1	189	1.75	0	69	20.40	3.50	138.51	216.56
Dong Nai	17	0.16	0	1	64306	595.43	97	3815	26.29	1.05	133.19	198.46
Dong Thap	10	0.09	0	1	29119	269.62	33	1216	27.20	1.06	66.71	84.76
Gia Lai	16	0.15	0	1	36601	338.90	0	3007	23.93	1.67	231.22	297.63
Ha Giang	15	0.14	0	1	65	0.60	0	16	19.79	4.61	122.54	159.96
Ha Nam	8	0.07	0	1	1157	10.71	0	361	23.64	4.73	10.63	17.54
Ha Tinh	19	0.18	0	1	1169	10.82	0	150	23.66	4.78	45.49	59.62
Hai Duong	10	0.09	0	1	995	9.21	0	180	23.80	4.68	88.36	94.60
Hai Phong	10	0.09	0	1	2727	25.25	0	325	23.57	4.80	22.36	32.28
Ha Noi	13	0.12	0	1	90948	842.11	1	13168	23.44	4.27	14.84	24.27
Hau Giang	8	0.07	0	1	3836	35.52	3	123	26.72	1.03	44.12	45.02
Ho Chi Minh City	66	0.61	0	1	216432	2004.00	116	9239	27.12	1.01	48.48	61.79
Hoa Binh	16	0.15	0	1	844	7.81	0	253	22.31	4.59	67.12	101.39
Hung Yen	6	0.06	0	1	1486	13.76	0	436	23.77	4.77	11.65	18.34
Khanh Hoa	18	0.17	0	1	50005	463.01	29	2452	24.27	2.01	87.95	80.44
Kien Giang	12	0.11	0	1	14595	135.14	9	582	26.98	0.97	148.30	149.36
Kon Tum	25	0.23	0	1	6905	63.94	0	810	21.66	1.55	215.70	233.16
Lai Chau	15	0.14	0	1	24	0.22	0	8	19.65	3.67	147.69	181.85
Lam Dong	27	0.25	0	1	4877	45.16	2	427	21.84	1.01	243.71	234.20
Lang Son	11	0.10	0	1	284	2.63	0	170	21.07	4.99	106.79	158.35
Lao Cai	35	0.32	0	1	221	2.05	0	65	19.63	4.42	100.57	106.71
Long An	17	0.16	0	1	28475	263.66	36	1130	27.22	1.06	95.36	118.40
Nam Dinh	15	0.14	0	1	6781	62.79	0	2194	24.05	4.65	16.52	25.99
Nghe An	28	0.26	0	1	2515	23.29	0	373	22.25	4.19	207.41	307.28
Ninh Binh	14	0.13	0	1	1106	10.24	0	311	23.69	4.66	15.42	25.20

Province	Flood exposure				Dengue count				Temperature (°C)		Precipitation (mm)	
	Total	Mean	Min	Max	Total	Mean	Min	Max	Mean	SD	Median	IQR
Ninh Thuan	25	0.23	0	1	4932	45.67	1	267	24.57	1.65	56.62	83.57
Phu Tho	12	0.11	0	1	1984	18.37	0	641	22.80	4.68	50.90	71.99
Phu Yen	24	0.22	0	1	19126	177.09	13	690	25.12	2.37	69.79	67.30
Quang Binh	26	0.24	0	1	16185	149.86	0	3590	23.32	3.69	120.59	130.40
Quang Nam	18	0.17	0	1	27538	254.98	0	3461	23.30	2.77	239.24	157.66
Quang Ngai	24	0.22	0	1	12311	113.99	3	901	24.33	2.78	118.10	91.56
Quang Ninh	15	0.14	0	1	1820	16.85	0	171	22.28	4.73	64.22	124.15
Quang Tri	18	0.17	0	1	9210	85.28	0	1442	23.96	3.16	77.82	70.54
Soc Trang	10	0.09	0	1	17631	163.25	16	526	26.81	0.97	83.65	91.66
Son La	20	0.19	0	1	241	2.23	0	138	20.39	3.92	165.60	278.26
Tay Ninh	7	0.06	0	1	17523	162.25	3	798	27.48	1.18	75.41	99.00
Thai Binh	12	0.11	0	1	1562	14.46	0	444	24.04	4.68	19.39	30.25
Thai Nguyen	17	0.16	0	1	666	6.17	0	168	22.37	4.74	48.98	72.15
Thanh Hoa	24	0.22	0	1	5282	48.91	0	1626	22.68	4.44	131.71	227.38
Thua Thien - Hue	26	0.24	0	1	6726	62.28	0	1102	23.96	2.99	125.57	84.39
Tien Giang	31	0.29	0	1	26070	241.39	34	1174	26.91	1.00	51.52	66.99
Tra Vinh	6	0.06	0	1	9458	87.57	4	478	26.83	0.96	53.99	60.31
Tuyen Quang	17	0.16	0	1	494	4.57	0	132	22.04	4.62	81.73	132.52
Vinh Long	32	0.30	0	1	11254	104.20	15	497	26.76	1.02	37.63	41.48
Vinh Phuc	6	0.06	0	1	1158	10.72	0	306	23.36	4.73	15.09	22.77
Yen Bai	31	0.29	0	1	433	4.01	0	123	20.59	4.40	122.36	166.85
National	1146	0.17	0	1	1129083	166.00	0	13168	24.05	4.02	153.30	191.4058

SD: standard deviation. IQR: inter quantile range.

Table S2. Summary of weekly time series data on flood exposure, dengue counts, weekly mean temperature, and weekly total precipitation over 7 years in Ho Chi Minh City for each district

District	Flood presence				Dengue count				Temperature (℃)		Precipitation (mm)	
	Total	Mean	Min	Max	Total	Mean	Min	Max	Mean	SD	Median	IQR
Binh Chanh	28	0.08	0	1	8335	22.84	3	109	27.12	1.18	34.46	52.77
Binh Tan	196	0.54	0	1	12679	34.74	2	155	27.14	1.18	33.39	52.73
Binh Thanh	128	0.35	0	1	6011	16.47	0	74	27.08	1.17	34.68	52.89
Can Gio	0	0	0	0	900	2.47	0	40	26.98	1.07	37.48	57.98
Cu Chi	0	0	0	0	3892	10.66	0	59	27.19	1.25	33.02	56.04
District 1	47	0.13	0	1	3618	9.91	0	40	27.08	1.17	34.66	52.91
District 10	9	0.02	0	1	3737	10.24	0	46	27.08	1.17	34.66	52.91
District 11	128	0.35	0	1	3755	10.29	0	35	27.13	1.18	33.75	52.07
District 12	124	0.34	0	1	7018	19.23	0	116	27.09	1.2	34.37	53.52
District 2	158	0.43	0	1	2711	7.43	0	53	27.01	1.17	36.11	52.84
District 3	3	0.01	0	1	3023	8.28	0	31	27.08	1.17	34.66	52.91
District 4	24	0.07	0	1	2740	7.51	0	28	27.08	1.17	34.66	52.91
District 5	68	0.19	0	1	2958	8.1	0	30	27.08	1.17	34.87	52.82
District 6	154	0.42	0	1	4054	11.11	1	39	27.12	1.18	34.27	52.88
District 7	90	0.25	0	1	5315	14.56	1	65	27.08	1.15	36.27	52.27
District 8	141	0.39	0	1	6999	19.18	2	62	27.09	1.17	34.47	53.15
District 9	84	0.23	0	1	5252	14.39	0	133	26.94	1.18	35.75	54.16
Go Vap	123	0.34	0	1	4249	11.64	0	54	27.09	1.18	34.44	52.49
Hoc Mon	38	0.1	0	1	6266	17.17	0	61	27.15	1.21	33.66	54.25
Nha Be	34	0.09	0	1	2109	5.78	0	30	27.05	1.14	36.94	55.33
Phu Nhuan	38	0.1	0	1	2213	6.06	0	28	27.08	1.17	34.66	52.91
Tan Binh	86	0.24	0	1	7228	19.8	1	91	27.12	1.17	33.92	52.17
Tan Phu	131	0.36	0	1	8513	23.32	0	134	27.17	1.18	33.21	52.95
Thu Duc	155	0.42	0	1	7590	20.79	0	89	27	1.19	34.76	53.70
HCMC	1987	0.23	0	1	121165	13.83	0	155	27.08	1.17	34.61	53.30

SD: standard deviation. IQR: inter quantile range

Table S3. Cross-comparison between ERA5-Land and WorldClim 2.1

Variable	N[a]	Mean [b]	r	R^2	MAE	p-value
**Temperature**	6,804	24.61°C	0.979	0.958	0.85 °C	< 0.001
**Precipitation**	6,804	149.44 mm	0.799	0.638	56.62 mm	< 0.001

a: Total number of province-month observations used in the validation.
b: mean value derived from the WorldClim 2.1 dataset.
r: Pearson correlation coefficient;
R^2: Coefficient of determination
MAE: Mean Absolute Error.

Table S4. Cumulative RRs of flood exposure across models at the national level

Model	Description	Model specification (forward stepwise)	Cumulative RR	Lower CI	Upper CI	p-value
1	Main model (dengue, flood, temporal factor, mean temperature, total precipitation, population size, and spatial factor)	$\log(Y_{it}) = a + cb(Flood_{it}, lag = 4, df = 3) + ns(Time_t, df = 3) + ns(Temp_{it}, df = 3) + ns(Precip_{it}, df = 3) + \log(Pop_{size_{it}}) + u_i$	1.25	1.00	1.56	0.050
2	+ Population density	$+ \beta_1 Pop_{dense_{it}}$	1.24	0.99	1.55	0.059
3	+ Poverty rate	$+ \beta_2 Poverty_{it}$	1.23	0.99	1.53	0.066
4	+ Income	$+ \beta_3 Income_{it}$	1.25	1.00	1.55	0.046
5	+ Agricultural land	$+ \beta_4 Agri_{land_{it}}$	1.25	1.00	1.56	0.047
6	+ Specially used land	$+ \beta_5 Special_{land_{it}}$	1.26	1.01	1.57	0.037
7	+ Homestead land	$+ \beta_6 Home_{land_{it}}$	1.26	1.01	1.57	0.038
8	+ Forestry land	$+ \beta_7 Forest_{it}$	1.28	1.02	1.59	0.030

Table S5. Cumulative RRs of flood exposure across models in HCMC

Model	Description	Model specification (forward stepwise)	Cumulative RR	Lower CI	Upper CI	p-value
1	Main model (dengue, flood, temporal factor, mean temperature, total precipitation, population size, and spatial factor)	$\mathbf{log}(\boldsymbol{Y_{it}}) = a + cb(Flood_{it}, lag = 15, df = 3) + ns(Time_t, df = 5) + ns(Temp_{it}, df = 3) + \log(Pop_{size_{it}}) + u_i$	1.10	1.04	1.17	0.002
2	+ Population density	$+ \beta_1 Pop_{dense_{it}}$	1.10	1.04	1.17	0.002
3	+ Poverty rate	$+ \beta_2 Poverty_{it}$	1.10	1.04	1.17	0.002
4	+ Income	$+ \beta_3 Income_{it}$	1.10	1.04	1.17	0.002

Table S6. Sensitivity analysis for different lag structures and degrees of freedom at the national level

	Lag=4, df=3/year			Lag=3, df=3/year			Lag=5, df=3/year			Lag=4, df=2/year			Lag=4, df=4/year		
	RR	Lower CI	Upper CI	RR	Lower CI	Upper CI	RR	Lower CI	Upper CI	RR	Lower CI	Upper CI	RR	Lower CI	Upper CI
Cumulative	1.25	1.00	1.56	1.29	1.06	1.57	1.27	0.99	1.63	1.05	0.83	1.33	1.25	1.00	1.56
Lag 0	0.99	0.89	1.08	0.98	0.89	1.09	1.01	0.92	1.11	0.97	0.87	1.07	0.99	0.90	1.09
Lag 1	1.08	1.02	1.15	1.09	1.01	1.18	1.06	1.00	1.12	1.01	0.95	1.08	1.08	1.02	1.15
Lag 2	1.13	1.05	1.21	1.12	1.04	1.21	1.09	1.02	1.16	1.04	0.96	1.12	1.12	1.04	1.21
Lag 3	1.07	1.02	1.14	1.07	0.96	1.18	1.08	1.02	1.15	1.03	0.97	1.10	1.07	1.01	1.13
Lag 4	0.97	0.88	1.07	-	-	-	1.04	0.98	1.09	1.01	0.91	1.12	0.97	0.88	1.07
Lag 5	-	-	-	-	-	-	0.98	0.89	1.07	-	-	-	-	-	-

Table S7. Sensitivity analysis for different lag structures and degrees of freedom at the HCMC level

	Lag=16, df=5/year			Lag=12, df=5/year			Lag=20, df=5/year			Lag=16, df=4/year			Lag=4, df=6/year		
	RR	Lower CI	Upper CI	RR	Lower CI	Upper CI	RR	Lower CI	Upper CI	RR	Lower CI	Upper CI	RR	Lower CI	Upper CI
Cumulative	1.096	1.027	1.170	1.101	1.037	1.170	1.122	1.047	1.204	1.100	1.030	1.171	1.091	1.023	1.164
Lag 0	0.980	0.964	0.996	0.976	0.957	0.994	0.983	0.969	0.998	0.972	0.956	0.988	0.985	0.968	1.001
Lag 1	0.987	0.974	1.000	0.985	0.971	0.998	0.988	0.976	1.000	0.981	0.968	0.994	0.990	0.977	1.003
Lag 2	0.994	0.984	1.003	0.994	0.984	1.003	0.992	0.983	1.002	0.990	0.980	1.000	0.996	0.986	1.005
Lag 3	1.000	0.993	1.007	1.002	0.994	1.010	0.997	0.989	1.004	0.998	0.991	1.006	1.001	0.993	1.008
Lag 4	1.006	0.999	1.012	1.009	1.000	1.018	1.001	0.995	1.007	1.006	1.000	1.013	1.005	0.999	1.012
Lag 5	1.011	1.004	1.018	1.015	1.005	1.025	1.005	1.000	1.010	1.013	1.006	1.020	1.009	1.002	1.016
Lag 6	1.015	1.007	1.023	1.019	1.008	1.030	1.009	1.003	1.014	1.019	1.011	1.027	1.012	1.004	1.021
Lag 7	1.018	1.009	1.027	1.021	1.010	1.031	1.012	1.005	1.018	1.023	1.014	1.032	1.015	1.006	1.024
Lag 8	1.019	1.010	1.029	1.021	1.012	1.030	1.014	1.007	1.021	1.025	1.015	1.034	1.016	1.007	1.025
Lag 9	1.019	1.011	1.028	1.019	1.011	1.027	1.016	1.008	1.024	1.025	1.016	1.034	1.016	1.007	1.025
Lag 10	1.018	1.010	1.026	1.016	1.007	1.026	1.017	1.009	1.025	1.023	1.015	1.031	1.015	1.007	1.023
Lag 11	1.015	1.008	1.023	1.013	0.999	1.027	1.017	1.009	1.025	1.019	1.012	1.026	1.013	1.006	1.020
Lag 12	1.012	1.005	1.019	1.009	0.991	1.028	1.017	1.009	1.024	1.014	1.008	1.021	1.010	1.004	1.017
Lag 13	1.008	1.000	1.015	-	-	-	1.015	1.009	1.022	1.008	1.001	1.016	1.007	0.999	1.015
Lag 14	1.003	0.993	1.013	-	-	-	1.014	1.008	1.019	1.001	0.992	1.011	1.003	0.993	1.013
Lag 15	0.997	0.984	1.010	-	-	-	1.011	1.006	1.017	0.994	0.981	1.007	0.999	0.986	1.013
Lag 16	0.992	0.975	1.009	-	-	-	1.008	1.002	1.015	0.987	0.971	1.003	0.995	0.978	1.012
Lag 17	-	-	-	-	-	-	1.005	0.998	1.013	-	-	-	-	-	-
Lag 18	-	-	-	-	-	-	1.002	0.992	1.012	-	-	-	-	-	-
Lag 19	-	-	-	-	-	-	0.999	0.986	1.011	-	-	-	-	-	-
Lag 20	-	-	-	-	-	-	0.995	0.980	1.010	-	-	-	-	-	-

Table S8. Comparison of spatial weighting methods for meteorological variable extraction

Main results	Type of weather data used in the main model	
	Area-weighted	Composite-weighted
Cumulative RR (0–4 months)	1.25 (1.00–1.56)	1.21 (0.97–1.52)
Peak lag (months)	2	2

Reference

1. Vietnam Ministry of Health. *Guidelines on the Regime of Declaration, Information, and Reporting of Infectious Diseases*. Hanoi, Vietnam, 31 December 2010.
2. Vietnam Ministry of Health. *Decision No. 1499/QĐ-BYT on the issuance of 'Guidelines for Surveillance and Prevention of Dengue Fever'*. Hanoi, Vietnam, Hanoi, Vietnam, 17 May 2011.
3. Vietnam Ministry of Health. *Decision No. 3711/QD-BYT on the issuance of 'Guidelines for Surveillance and Prevention of Dengue Fever'*. Hanoi, Vietnam, 19 September 2014.
4. Vietnam Ministry of Health. *Decision No. 458/QĐ-BYT on the issuance of guidelines for the diagnosis and treatment of dengue fever*. Hanoi, Vietnam, 16 February 2011.
5. Harris I, Osborn TJ, Jones P, et al. Version 4 of the CRU TS monthly high-resolution gridded multivariate climate dataset. *Sci Data* 2020; 7: 109.
6. Fick SE, Hijmans RJ. WorldClim 2: new 1-km spatial resolution climate surfaces for global land areas. *International Journal of Climatology* 2017; 37: 4302–4315.
7. International Monetary Fund. *Staff Report for the 2023 Article IV Consultation-Informational Annex: Vietnam*. Washington, D.C, 2023.
8. United Nations. Country Profile: Viet Nam. *UNdata*, https://data.un.org/CountryProfile.aspx/en/CountryProfile.aspx?crName=Viet%20Nam (accessed 19 April 2025).
9. World Bank Group. *Macro Poverty Outlook for East Asia and the Pacific: Country-by-country analysis and projections for the developing world*. Washington DC, 2024.
10. General Statistics Office. *Decision No. 1006/QĐ-TCTK dated May 20, 2020 on functions, tasks, powers, and organizational structure of provincial and municipal statistics offices under the central government*. Ha Noi, Vietnam, May 2020.
11. Schmidt W-P, Suzuki M, Dinh Thiem V, et al. Population Density, Water Supply, and the Risk of Dengue Fever in Vietnam: Cohort Study and Spatial Analysis. *PLoS Med* 2011; 8: e1001082.
12. Sajib AH, Akter S, Saha G, et al. Demographic-environmental effect on dengue outbreaks in 11 countries. *PLoS One* 2024; 19: e0305854.
13. Gao P, Pilot E, Rehbock C, et al. Land use and land cover change and its impacts on dengue dynamics in China: A systematic review. *PLoS Negl Trop Dis* 2021; 15: e0009879.
14. Zhang Y, Riera J, Ostrow K, et al. Modeling the relative role of human mobility, land-use and climate factors on dengue outbreak emergence in Sri Lanka. *BMC Infect Dis* 2020; 20: 649.
15. Feng B, Zhang Y, Bourke R. Urbanization impacts on flood risks based on urban growth data and coupled flood models. *Natural Hazards* 2021; 106: 613–627.
16. Son CH, Lee CH, Ban YU. Analysis of the impact and moderating effect of high-density development on urban flooding. *Heliyon* 2023; 9: e22695.
17. Smith J. Dengue and its correlation to poverty: a scoping review. *South Florida Journal of Health* 2025; 6: e5123.

18. Bavia L, Melanda FN, de Arruda TB, et al. Epidemiological study on dengue in southern Brazil under the perspective of climate and poverty. *Sci Rep* 2020; 10: 2127.

19. Hashizume M, Dewan AM, Sunahara T, et al. Hydroclimatological variability and dengue transmission in Dhaka, Bangladesh: A time-series study. *BMC Infect Dis*; 12. Epub ahead of print 24 April 2012. DOI: 10.1186/1471-2334-12-98.

20. Lowe R, Gasparrini A, Van Meerbeeck CJ, et al. Nonlinear and delayed impacts of climate on dengue risk in Barbados: A modelling study. *PLoS Med* 2018; 15: e1002613.

21. Kim T Van, Pham TND, Do B, et al. Low HBV knowledge is associated with low HBV vaccination uptake in general adult population despite incentivization of HBV vaccination. *BMC Infect Dis* 2024; 24: 470.
